# Investigating the Role of Metabolic Bariatric Surgery in Achieving Remission of Type 2 Diabetes Mellitus and Evaluating the Effects on Glycemic Control and Metabolic Health

**DOI:** 10.7759/cureus.86539

**Published:** 2025-06-22

**Authors:** Masseh Ahmad Ahmadi, Muhammad Dewan Ojla, Maheen Iqbal, Ayman Tahir, Noor Un Nahar, Muhammad Hamza, Hajra Amin

**Affiliations:** 1 Internal Medicine, Shalamar Hospital, Lahore, PAK; 2 Internal Medicine, Fauji Foundation Hospital, Rawalpindi, PAK; 3 Internal Medicine, Allama Iqbal Teaching Hospital, Dera Ghazi Khan, PAK; 4 Internal Medicine, Lahore General Hospital, Lahore, PAK; 5 Internal Medicine, Pakistan Kidney and Liver Institute, Lahore, PAK; 6 General Surgery, Muzaffarabad General Hospital, Muzaffarabad, PAK

**Keywords:** bariatric surgery, glycemic control, metabolic health, obesity, type 2 diabetes

## Abstract

Background

Type 2 diabetes mellitus (T2DM) is a prevalent metabolic disorder often associated with obesity. Despite conventional management strategies, many patients fail to achieve sustained remission.

Objective

This study aimed to investigate the role of metabolic bariatric surgery (MBS), specifically Roux-en-Y gastric bypass (RYGB) and sleeve gastrectomy (SG), in achieving remission of T2DM and evaluating the effects on glycemic control and overall metabolic health in patients with obesity.

Methods

This retrospective study was conducted at Shalamar Hospital, Lahore, Pakistan from January 2021 to December 2024. Data for this study were collected from electronic medical records. It included glycated hemoglobin* (*HbA1c) levels at baseline and subsequent intervals of three, six, and 12 months. Comparative analyses between the RYGB and SG groups were performed. Fasting blood glucose levels, use of antidiabetic medications (including both oral hypoglycemic agents and insulin), and anthropometric measurements such as weight and BMI were also tracked over time. To identify independent predictors of complete diabetes remission at 12 months, multivariate logistic regression analysis was performed. Results were reported as ORs with 95% CIs and Wald chi-square statistics. A p-value of <0.05 was considered statistically significant for all tests.

Results

At baseline, the mean age was 47.2 years, and the mean BMI was 42.6 kg/m². At three months, 38 of 81 (46.9%) patients achieved early glycemic response of T2DM. At six months, 21 of 81 (25.9%) showed partial response. By 12 months, complete remission was observed in 29 of 81 patients (35.8%). RYGB patients had higher rates than SG patients at both three months (25/43 (58.1%) vs. 13/38 (34.2%)) and 12 months (20/43 (46.5%) vs. 9/38 (23.7%)).

Conclusions

MBS, particularly RYGB, offers a highly effective treatment for T2DM in patients with obesity, with significant and sustained improvements in glycemic control and metabolic health. Early intervention, particularly in patients with shorter diabetes duration and lower preoperative HbA1c, maximizes the likelihood of achieving remission.

## Introduction

Type 2 diabetes mellitus (T2DM) is a chronic and progressive metabolic disorder that has reached epidemic levels globally, affecting millions of individuals and imposing a significant burden on healthcare systems worldwide [1].

The main features of the disease include insulin resistance as cells resist insulin effects and a progressive decline in insulin-producing pancreatic β-cell function [2]. The presence of obesity in T2DM worsens insulin resistance throughout the body and speeds up β-cell function reduction in the pancreas. The approved treatment approaches for T2DM, including lifestyle adjustments and oral hypoglycemics, together with insulin therapy, do not sustain remission or produce optimal glycemic results in many patients [3,4]. The multiple serious diabetes complications that arise from uncontrolled blood sugar levels underscore why better treatment solutions are needed [5].

Metabolic bariatric surgery (MBS) or weight-loss surgery has received more attention, as it proves to be an efficient method of treating people with obesity and T2DM. The original purpose of MBS aimed to assist patients with obesity in achieving major weight loss, but recent research indicates it extensively helps control blood sugar levels, resulting in T2DM remission for selected patient groups [6]. MBS provides additional metabolic advantages that extend past weight reduction, thus indicating that independent mechanisms contribute to better insulin sensitivity and enhanced glucose metabolism [7]. The study outcomes about MBS for T2DM management have created essential debates regarding its use as a first-choice treatment for patients whose standard treatments have failed to work.

The three major MBS approaches feature different operation methods, specifically Roux-en-Y gastric bypass (RYGB), sleeve gastrectomy (SG), and adjustable gastric banding. Research has demonstrated that RYGB creates highly efficient glycemic control and elevated rates of T2DM remission among other surgical choices [8]. Although the precise mechanisms underlying T2DM remission after MBS are not fully understood, growing evidence suggests that changes in gastrointestinal hormones play a central role. Notably, there is a marked postprandial increase in glucagon-like peptide-1 (GLP-1), along with changes in peptide YY and suppression of ghrelin, which collectively enhance insulin secretion and sensitivity. Among these, the elevated postprandial GLP-1 response appears to be the most prominent and consistent mechanism driving glycemic improvement after RYGB [9]. These hormonal changes, in combination with altered gut nutrient sensing and microbiota, contribute significantly to improved glucose homeostasis independent of weight loss. Medical research demonstrates that patients often experience a fast decrease in glucose levels after MBS before substantial weight reduction, showing that the surgery's metabolic effects may function independently of weight loss [10,11].

The potential impacts of MBS on T2DM remission, together with metabolic health, remain unclear, even though short-term outcomes show promise. The improvement of glycemic control, which researchers have observed in postoperative patients, continues to face doubts regarding its sustained effectiveness. The evidence shows conflicting data regarding diabetes symptoms relapse among weight loss surgery recipients, yet it demonstrates long-term remission achievements in many patients [12,13]. MBS presents extended safety risks, which include nutritional deficits in addition to gastrointestinal problems and weight regain potential. Knowledge about the extended metabolic effects on MBS and its ability to maintain T2DM remission and its influence on metabolic health must guide the choice of using surgical treatment for T2DM management. The surgical procedure yields dual benefits through its improvement of blood sugar control and favorable modification of blood lipid levels, blood pressure (BP) measurements, and inflammatory marker levels. Postsurgical patient improvements in cardiovascular disease risk factors help generate better health outcomes among these patients [14].

Objective

This study aimed to investigate the role of MBS in achieving remission of T2DM and to evaluate the effects on glycemic control and overall metabolic health in patients with obesity.

## Materials and methods

Study design and setting

This retrospective study was conducted at Shalamar Hospital, Lahore, Pakistan, including patients who underwent bariatric surgery between January 2021 and December 2023, allowing a minimum follow-up period of 12 months. Data collection was completed by December 2024.

Inclusion and exclusion criteria

Participants were required to be between 18 and 65 years of age, have a confirmed diagnosis of T2DM for a minimum of one year before undergoing surgery, and have undergone either RYGB or SG. Additionally, participants must have completed at least 12 months of postoperative follow-up.

Diagnosis of T2DM was based on American Diabetes Association (ADA) criteria, which include fasting plasma glucose (FPG) ≥126 mg/dL (7.0 mmol/L), two-hour plasma glucose ≥200 mg/dL (11.1 mmol/L) during a 75 g oral glucose tolerance test, glycated hemoglobin (HbA1c) ≥6.5% (48 mmol/mol), or a random plasma glucose ≥200 mg/dL (11.1 mmol/L) in a patient with classic symptoms of hyperglycemia [15].

Individuals were excluded if they had a diagnosis of type 1 diabetes or latent autoimmune diabetes in adults, had undergone previous MBS, were using corticosteroids or other medications known to significantly affect glucose metabolism, e.g., atypical antipsychotics (olanzapine and risperidone), thiazide diuretics, beta-blockers, or immunosuppressive agents (tacrolimus and cyclosporine), had incomplete medical records, or were lost to follow-up before reaching the 12-month postoperative mark.

Data collection

Data for this study were collected from electronic medical records of individuals who underwent MBS (either SG or RYGB). It included HbA1c levels at baseline and subsequent intervals of three months, six months, and 12 months. Fasting blood glucose following a minimum of 8 hours of overnight fasting alongside antidiabetic medication usage (oral hypoglycemic agents and insulin) and measures of weight and BMI remained subjects of evaluation throughout the study duration. The impact of surgery on overall metabolism was evaluated through assessments of liver function tests, together with BP measurements and lipid profile assessments. The ADA criteria defined complete remission as FPG <100 mg/dL and HbA1c <5.7%, sustained for at least 1 year without the use of any glucose-lowering medications, and partial remission was defined as fasting glucose between 100 and 125 mg/dL and HbA1c <6.5%, sustained for at least 1 year without antidiabetic medications [16].

Postoperative care and nutritional protocols

The patients received postoperative care by following standardized nutritional protocols with vitamin supplementation and needing regular consultations with both endocrinologists and dietitians [17]. The patients received guidance for structured dietary planning and physical activity, and their glycemic and metabolic status were evaluated regularly during follow-up sessions. Outcomes were explicitly compared between RYGB and SG groups to evaluate procedure-specific efficacy.

Stage 1 (Week 1-2): Clear liquids only, including broth, sugar-free gelatin, and electrolyte solutions. Stage 2 (Week 3-4): Full liquids, including protein shakes (≥60 g/day protein), lactose-free milk, and blended soups. Stage 3 (Week 5-6): Soft, pureed foods introduced gradually. Stage 4 (after Week 6): Transition to regular solid foods, with focus on high-protein, low-carbohydrate, low-fat meals. Patients were encouraged to eat small, frequent meals and chew thoroughly.

All patients received daily multivitamin supplementation, along with calcium (1200-1500 mg), vitamin D (3000 IU), vitamin B12 (350-500 mcg/week orally or intramuscularly), and iron supplements (as needed).

Surgical technique

All surgeries were performed by experienced bariatric surgeons, using standardized laparoscopic techniques. For RYGB, a small gastric pouch (~30 mL) was created, followed by an antecolic-antegastric Roux-en-Y configuration with a 50 cm biliopancreatic limb and a 150 cm alimentary (Roux) limb. SG was performed by resecting approximately 75%-80% of the stomach along a 36 Fr bougie, beginning 4-6 cm from the pylorus and extending to the angle of His, including complete fundus resection. Staple line reinforcement was performed selectively. All patients received intraoperative leak testing and postoperative monitoring per institutional protocol.

The decision to perform SG or RYGB was individualized based on patient characteristics, clinical profile, multidisciplinary team recommendations, and International Federation for the Surgery of Obesity and Metabolic Disorders (IFSO)/American Society of Metabolic and Bariatric Surgery (ASMBS) standards [18]. RYGB was preferred for patients with long-standing T2DM (>5 years), higher baseline HbA1c (>8.5%), presence of gastroesophageal reflux disease (GERD), and poor glycemic control on multiple agents or insulin. SG was selected for patients with BMI <45 kg/m² who had no GERD, higher surgical risk due to comorbidities, and preference for SG after counseling on risks and benefits.

Statistical analysis

Data were analyzed using Statistical Package for Social Sciences (SPSS) version 26 (IBM Corp., Armonk, NY). Continuous variables were summarized using means and SDs, while categorical variables were presented as counts and percentages (n (%)). Paired t-tests were used to assess changes in continuous variables such as HbA1c, fasting glucose, BMI, systolic BP, and low-density lipoprotein (LDL)cholesterol between baseline and follow-up time points (six and 12 months). For categorical variables (e.g., medication usage AND remission status), chi-square (χ²) tests were used to compare distributions over time and between surgical groups (RYGB vs. SG).

Group-wise comparisons of baseline characteristics were conducted using independent t-tests for continuous variables and chi-square tests for categorical variables to ensure comparability between the RYGB and SG groups. To identify factors independently associated with complete diabetes remission at 12 months, a multivariate logistic regression model was constructed. Predictor variables included age, duration of diabetes, preoperative HbA1c, baseline BMI, preoperative insulin use, surgery type, and percentage of weight loss at six months. Results were expressed as ORs with 95% CIs, accompanied by Wald chi-square statistics.

Model diagnostics included checks for multicollinearity using variance inflation factors (VIFs), and model fit was assessed using the Hosmer-Lemeshow goodness-of-fit test. A two-tailed p-value of <0.05 was considered statistically significant for all analyses.

## Results

The mean age of all patients was 47.2±8.5 years (n=81), with the RYGB group being slightly younger at 46.8±8.2 years (n=43) and the SG group slightly older at 47.6±8.8 years (n=38). The mean BMI was 42.6±5.2 kg/m² for all patients (n=81), with RYGB patients having a mean BMI of 43.1±5.4 kg/m² (n=43) and SG patients having a slightly lower mean BMI of 42.0±4.9 kg/m² (n=38). Diabetes duration was relatively similar across all groups, with a mean of 8.3±3.6 years (n=81) for all patients, 8.1±3.3 years (n=43) for the RYGB group, and 8.6±4.0 years (n=38) for the SG group. Baseline HbA1c levels were 8.7±1.2% for all patients (n=81), 8.8±1.1% for RYGB patients (n=43), and 8.6±1.3% for SG patients (n=38) (Table 1).

**Table 1:** Baseline characteristics of study participants Data were represented as mean ± SD. Significance level: p<0.05. An independent t-test was used for continuous variables (e.g., age, BMI, and HbA1c), and a chi-square test was used for categorical variables (e.g., gender and medication use). BMI, body mass index; FBG, fasting blood glucose; HbA1c, glycated hemoglobin; RYGB, Roux-en-y gastric bypass; SG, sleeve gastrectomy

Variable	All patients (n=81)	RYGB (n=43)	SG (n=38)	p-value
Mean age (years)	47.2±8.5	46.8±8.2	47.6±8.8	0.62
Mean BMI (kg/m²)	42.6±5.2	43.1±5.4	42.0±4.9	0.38
Diabetes duration (years)	8.3±3.6	8.1±3.3	8.6±4.0	0.55
Baseline HbA1c (%)	8.7±1.2	8.8±1.1	8.6±1.3	0.41
Baseline FBG (mg/dL)	168.5±24.3	170.2±25.1	166.7±23.4	0.48

At three months, 38 (46.9%) patients achieved complete remission, with 25 (58.1%) in the RYGB group and 13 (34.2%) in the SG group (p=0.018). At six months, partial remission was observed in 21 (25.9%) patients, with 10 (23.3%) in the RYGB group and 11 (28.9%) in the SG group (p=0.250). By 12 months, 29 (35.8%) patients achieved complete remission, with 20 (46.5%) in the RYGB group and 9 (23.7%) in the SG group (p=0.026) (Table 2).

**Table 2:** Early glycemic responses and 12-month remission rates of type 2 diabetes mellitus following bariatric surgery Values were expressed as n (%). Significance level: p<0.05. The chi-square test was used to compare categorical remission outcomes between groups. RYGB, Roux-en-y gastric bypass; SG, sleeve gastrectomy

Outcome status	All patients (n=81)	RYGB (n=43)	SG (n=38)	p-value (χ²)
Early glycemic response (3 months)	38 (46.9%)	25 (58.1%)	13 (34.2%)	0.018 (χ² = 5.61)
Partial response (3 months)	14 (17.3%)	7 (16.3%)	7 (18.4%)	0.812 (χ²=0.06)
Sustained glycemic response (6 months)	33 (40.7%)	21 (48.8%)	12 (31.6%)	0.102 (χ²=2.68)
Partial response (6 months)	21 (25.9%)	10 (23.3%)	11 (28.9%)	0.552 (χ²=0.36)
Complete remission (12 months)	29 (35.8%)	20 (46.5%)	9 (23.7%)	0.026 (χ²=4.95)
Partial remission (12 months)	18 (22.2%)	9 (20.9%)	9 (23.7%)	0.761 (χ²=0.09)

At baseline, patients in both groups had comparable metabolic profiles. The mean HbA1c was 8.8% in the RYGB group and 8.6% in the SG group. Fasting glucose levels were 170.2 mg/dL and 166.7 mg/dL, respectively, while BMI averaged 43.1 kg/m² for RYGB group and 42.0 kg/m² for SG group. At six months, the RYGB group showed a more pronounced improvement in HbA1c (5.7% vs. 6.2%) and fasting glucose (92.1 mg/dL vs. 98.5 mg/dL) compared to SG group. Similarly, BMI reduction was greater in RYGB (34.2 vs. 36.4 kg/m²), as was systolic BP reduction (13.5 mmHg vs. 10.6 mmHg). LDL cholesterol reductions were also observed in both groups (18.9 mg/dL in RYGB vs. 16.5 mg/dL in SG). At 12 months, the RYGB group maintained lower HbA1c (5.8% vs. 6.4%) and fasting glucose (91.5 mg/dL vs. 106.2 mg/dL), along with lower BMI (33.8 kg/m² vs. 36.0 kg/m²) and greater reductions in systolic BP (15.2 mmHg vs. 12.7 mmHg) and LDL (21.4 mg/dL vs. 16.0 mg/dL) (Table 3).

**Table 3:** Metabolic outcomes over time Data were shown as mean ± SD. Paired t-tests were used for within-group comparisons, and independent t-tests were used for between-group comparisons. BMI, body mass index; BP, blood pressure; HbA1c, glycated hemoglobin; LDL, low-density lipoprotein; RYGB, Roux-en-y gastric bypass; SG, sleeve gastrectomy

Time point	Outcome	RYGB (n=43)	SG (n=38)	p-value (between groups)
HbA1c (%)	Baseline	8.8±1.1	8.6±1.3	0.41
	6 months	5.7±0.8 (t=14.3, p<0.001)	6.2±0.9 (t=10.1, p<0.001)	0.010
	12 months	5.8±0.9 (t=13.0, p<0.001)	6.4±1.0 (t=11.2, p<0.001)	0.003
Fasting glucose (mg/dL)	Baseline	170.2±25.1	166.7±23.4	0.48
	6 months	92.1±11.8 (t=15.6, p<0.001)	98.5±13.1 (t=11.4, p<0.001)	0.018
	12 months	91.5±10.4 (t=14.1, p<0.001)	106.2±14.1 (t=10.9, p<0.001)	0.001
BMI (kg/m²)	Baseline	43.1±5.4	42.0±4.9	0.27
	6 months	34.2±4.6 (t=16.5, p<0.001)	36.4±4.5 (t=13.2, p<0.001)	0.038
	12 months	33.8±4.8 (t=15.8, p<0.001)	36.0±5.2 (t=12.7, p<0.001)	0.042
Systolic BP reduction (mmHg)	Baseline	0.0±0.0	0.0±0.0	—
	6 months	13.5±4.9 (t=13.7, p<0.001)	10.6±4.4 (t=10.5, p<0.001)	0.021
	12 months	15.2±5.0 (t=14.8, p<0.001)	12.7±5.4 (t=11.2, p<0.001)	0.041
LDL reduction (mg/dL)	Baseline	0.0±0.0	0.0±0.0	—
	6 months	18.9±9.4 (t=12.5, p<0.001)	16.5±8.9 (t=10.1, p<0.001)	0.150
	12 months	21.4±8.9 (t=13.1, p<0.001)	16.0±9.2 (t=10.3, p<0.001)	0.022

At baseline, insulin use was reported in 29 (67.4%) patients in the RYGB group and 24 (63.2%) patients in the SG group. Oral hypoglycemic agent usage was also similar, with 36 patients (83.7%) in the RYGB group and 30 (78.9%) patients in the SG group, and none were medication free. At six months, insulin usage dropped to seven (16.3%) patients in the RYGB group compared to 10 (26.3%) patients in the SG group. Use of oral agents declined to 10 (23.2%) patients in the RYGB group and 13 (34.2%) patients in the SG group. Notably, 26 (60.5%) patients in the RYGB group became medication free compared to 16 (42.1%) patients in the SG group. By 12 months, insulin use remained lower in the RYGB (eight patients, 18.6%) group than the SG (13 patients, 34.2%) group, and more patients remained off all medications in the RYGB (24 patients, 55.8%) group compared to the SG (11 patients, 28.9%) group (Table 4).

**Table 4:** Medication usage over time Values were expressed as n (%). Significance level: p<0.05. The chi-square tests were performed between RYGB and SG groups at each time point. NA, not available; RYGB, Roux-en-y gastric bypass; SG, sleeve gastrectomy

Time point	Group	Insulin use, n (%)	Oral agent use, n (%)	Medication free, n (%)	p-value, insulin (χ²)	p-value, oral (χ²)	p-value, medication free (χ²)
Baseline	RYGB	29 (67.4%)	36 (83.7%)	0 (0.0%)	NA	NA	NA
	SG	24 (63.2%)	30 (78.9%)	0 (0.0%)	0.72	0.58	NA
6 months	RYGB	7 (16.3%)	10 (23.2%)	26 (60.5%)	<0.001 (χ²=18.2)	<0.001 (χ²=20.6)	<0.001 (χ²=24.8)
	SG	10 (26.3%)	13 (34.2%)	16 (42.1%)			
12 months	RYGB	8 (18.6%)	11 (25.6%)	24 (55.8%)	0.003 (χ²=9.0)	0.006 (χ²=8.2)	0.011 (χ²=10.5)
	SG	13 (34.2%)	15 (39.5%)	11 (28.9%)			

Multivariate logistic regression analysis was conducted to identify independent predictors of T2DM remission at 12 months. Key variables included preoperative characteristics and early postoperative weight loss. Significant predictors included diabetes duration <8 years (OR=2.8, 95% CI: 1.5-5.1, χ²=9.59, p=0.002), preoperative HbA1c <8.0% (OR=1.9, 95% CI: 1.1-3.2, χ²=6.43, p=0.011), undergoing RYGB (vs. SG; OR=2.3, 95% CI: 1.3-4.0, χ²=5.36, p=0.021), and weight loss ≥20% at six months (OR=2.6, 95% CI: 1.4-4.8, χ²=8.12, p=0.004). Absence of preoperative insulin use was also associated with higher odds of remission (OR=2.0, 95% CI: 1.1-3.7, χ²=5.99, p=0.014). Although younger age and lower BMI showed a trend toward better outcomes, they did not reach statistical significance. No multicollinearity was observed (all VIF <2), and the model had good fit (Hosmer-Lemeshow test, p=0.38) with R²=0.427 (Table 5).

**Table 5:** Predictors of type 2 diabetes mellitus remission (multivariate analysis) ORs with 95% CIs were reported. The significance level was set at p<0.05. Multivariate logistic regression was used to assess independent predictors of diabetes remission at 12 months, and Wald chi-square statistics were reported. BMI, body mass index; HbA1c, glycated hemoglobin; RYGB, Roux-en-y gastric bypass; SG, sleeve gastrectomy

Predictor	Category	Reference group	OR	95% CI	Wald χ²	p-value
Duration of diabetes	<8 years	≥8 years	2.8	1.5–5.1	9.59	0.002
Preoperative HbA1c	<8.0%	≥8.0%	1.9	1.1–3.2	6.43	0.011
Surgery type	RYGB	SG	2.3	1.3–4.0	5.36	0.021
Age	<50 years	≥50 years	1.5	0.9–2.6	2.63	0.105
Baseline BMI	<45 kg/m²	≥45 kg/m²	1.3	0.8–2.3	1.55	0.213
Preoperative insulin use	No	Yes	2.0	1.1–3.7	5.99	0.014
≥20% weight loss at 6 months	Yes	No	2.6	1.4–4.8	8.12	0.004

## Discussion

This study demonstrates that MBS is a highly effective intervention for achieving significant and sustained improvements in glycemic control and metabolic health in patients with T2DM. The research results show that MBS works as an effective metabolic therapy because early glycemic response was reached by 46% of patients at three months, while more than one-third of patients maintained remission status for 12 months.

The data on remission rates between RYGB and SG patients follow existing research data [19]. Glycemic outcomes demonstrated superior performance from RYGB because 46.5% of patients achieved complete remission at the 12-month follow-up compared to 23.7% in the SG group. The significant modifications in incretin production and insulin sensitivity, together with gut-brain signaling alterations that result from RYGB, probably explain these findings [20]. All patients experienced considerable drops in their HbA1c numbers, which started at baseline 8.7%, reached 5.9% at six months, and stabilized at 6.1% during the 12-month follow-up. Long-term patients with diabetes have demonstrated sustained better blood sugar regulation, yet their results may exhibit slight worsening over time, especially when their diabetes has endured for longer durations [21]. Postoperative lifestyle support and metabolic monitoring demonstrate their importance because remission relapses continue to appear in patients [22]. The overall remission rate observed in our cohort was slightly lower than those reported in other studies. One likely explanation is the relatively long preoperative diabetes duration in our patient population, with a mean of 8.3 years. Prior research has shown that longer diabetes duration (>8 years) is associated with diminished β-cell reserve and irreversible pancreatic damage, thereby reducing the likelihood of achieving complete remission following surgery. This aligns with our multivariate analysis, which demonstrated several factors that significantly influenced diabetes remission after bariatric surgery. Duration of diabetes <8 years was a strong predictor, supporting evidence that prolonged hyperglycemia results in irreversible pancreatic β-cell failure. Preoperative HbA1c <8.0% and lack of insulin requirement reflected better residual insulin production, which predicted higher remission. The type of surgery was critical: patients undergoing RYGB were 2.3 times more likely to achieve remission than those receiving SG, consistent with known hormonal and metabolic advantages of bypass procedures. Additionally, early weight loss ≥20% by six months independently increased remission likelihood. These findings emphasize the importance of patient selection, early surgical intervention, and achieving robust weight loss postoperatively.

The study documented substantial clinical development of multiple metabolic factors through reductions in fasting glucose, together with LDL cholesterol, systolic BP, and weight measurements. The evidence shows that patients kept a substantial mean weight loss of 25.9 kg at 12 months, and their BMI decreased by more than 8 points, demonstrating the lasting impact of surgical weight loss. The important metabolic breakthroughs are vital because T2DM carries a strong connection to cardiovascular risks. Medication use significantly declined after surgery. At the six-month follow-up, 50% of patients did not need medication, but by the 12-month follow-up, 42% of patients remained medication free. MBS enables a substantial number of patients to recover endogenous glucose regulation to a degree that can diminish or halt their need for medication, according to findings in Ghanem et al.'s study [23]. In addition to its metabolic benefits, RYGB carries potential long-term complications that require careful postoperative management. Common issues include nutritional deficiencies (iron, vitamin B12, calcium, folate, and fat-soluble vitamins) due to malabsorption, which may result in anemia, osteoporosis, or neuropathies if unaddressed. Another critical yet underrecognized complication is postprandial hyperinsulinemic hypoglycemia (PHH), particularly in patients with pre-existing T2DM, caused by exaggerated insulin responses from elevated GLP-1 levels. This can lead to neuroglycopenic symptoms, including confusion and syncope. A recent cohort study underscores the clinical significance of PHH after RYGB [24]. These risks highlight the necessity for long-term metabolic follow-up, micronutrient monitoring, and structured dietary counseling, which must be considered when discussing surgical options with patients.

This study has several limitations. First, its retrospective design introduces potential selection bias and depends on the accuracy of medical records. Second, while the 12-month follow-up provides valuable short-term insights, longer-term data are needed to assess the durability of remission and metabolic improvements. Third, the relatively small sample size limits subgroup analysis and may reduce the generalizability of findings. Fourth, nutritional deficiencies and postprandial hypoglycemia, particularly relevant in RYGB patients, were not assessed, which limits our understanding of long-term safety. Additionally, although we included patients with varying durations of diabetes, existing evidence suggests that a duration exceeding 8 years is associated with irreversible β-cell dysfunction, potentially reducing remission rates. Given that the mean diabetes duration in our cohort was 8.3 years, this may have impacted outcomes and underscores the need for earlier surgical intervention in appropriate candidates.

## Conclusions

MBS, particularly RYGB, is an effective treatment for T2DM in patients with obesity, with superior outcomes compared to SG. At 12 months, approximately one-third (35.8%) of patients achieved complete remission, with higher rates observed in those undergoing RYGB than SG. However, the modest remission rate may be attributed to the cohort’s long diabetes duration (>8 years), highlighting the importance of early surgical intervention. While MBS improves metabolic health, long-term follow-up remains essential to monitor nutritional deficiencies and late complications such as postbariatric hypoglycemia.
